# Pseudo-SAH in a patient with methanol poisoning: a case report

**DOI:** 10.1186/s13256-026-06118-4

**Published:** 2026-05-20

**Authors:** Sahar Defaee, Elham Ouspid, Elham Barahimi, MohammadHosein Sheybani-Arani, Fatemeh Khajavi-Mayvan

**Affiliations:** 1 Infectious and Tropical Diseases Research Center, Hormozgan University of Medical Sciences, Bandar Abbas, Iran; 2 Department of Neurology, Faculty of Medicine, Hormozgan University of Medical Sciences, Bandar Abbas, Iran; 3 Infectious and Tropical Diseases Research Center, Hormozgan University of Medical Sciences, BandarAbbas, Iran; 4 Clinical Research Development Center of Mohammadi Hospital, Hormozgan University of Medical Sciences, Bandar Abbas, Iran; 5 Student Research Committee, Faculty of Medicine, Bandar Abbas, Hormozgan Iran

**Keywords:** Pseudo-SAH, Methanol, Poisoning

## Abstract

**Background:**

Methanol is a toxic alcohol found in many industrial products. Ingestion can cause severe metabolic acidosis, optic nerve damage, and central nervous system depression. While bilateral putaminal necrosis is a typical radiologic finding, pseudo-subarachnoid hemorrhage (pseudo-SAH) is a rare and often misdiagnosed complication. Prompt recognition is essential to avoid unnecessary interventions.

**Case presentation:**

A 31-year-old Caucasian Iranian male presented unresponsive after consuming 1–2 L of homemade methanol. He experienced cardiac arrest upon arrival but was successfully resuscitated. Laboratory tests showed severe metabolic acidosis, acute kidney injury and hepatic enzymes elevation. Hemodialysis and supportive care were initiated. Despite treatment, the patient remained comatose. On day 3, neurological examination revealed fixed pupils and absent reflexes. Brain CT showed diffuse cerebral edema and hyperdensities initially misinterpreted as subarachnoid hemorrhage. Following neurology consultation, these findings were identified as pseudo-SAH, reflecting severe hypoxic–ischemic brain injury. The patient was declared brain dead following evidence of cerebral herniation.

**Conclusion:**

Pseudo-SAH is a radiologic manifestation of severe cerebral edema rather than a primary cause of death. In methanol intoxication, it can mimic true subarachnoid hemorrhage on imaging and lead to diagnostic confusion. Clinicians should maintain a high index of suspicion for pseudo-SAH in patients with methanol poisoning and unexplained radiologic findings to ensure accurate diagnosis and appropriate management.

## Introduction

Methanol, a highly toxic alcohol, possesses olfactory and gustatory characteristics similar to ethanol [1, 2]. It is a constituent of various industrial and consumer products, including varnishes, paint removers, perfumes, antifreeze, copy machine fluid, and gasoline mixtures, thereby posing a risk of accidental or intentional ingestion. As a central nervous system (CNS) depressant, methanol exhibits potential toxicity following ingestion, inhalation, or transdermal exposure [3].

Acute methanol intoxication, frequently resulting from accidental ingestion, induces metabolic acidosis through the production of formic acid and lactic acid, alongside a spectrum of CNS disturbances ranging from inebriation and drowsiness to obtundation, seizure, and coma. The selective toxicity of methanol toward the optic nerve and basal ganglia is a well-established feature. Radiologically, severe methanol toxicity often manifests as bilateral putaminal necrosis and diffuse white matter involvement, typically culminating in death within 3 days. True subarachnoid hemorrhage is rare, whereas pseudo-subarachnoid hemorrhage (pseudo-SAH) may occur secondary to profound cerebral edema [4–7].

Without therapeutic intervention, ingestion of 30 ml of pure methanol typically results in fatality, while as little as 4 ml can induce blindness. Blood methanol levels exceeding 200 mg/L are considered toxic, and levels surpassing 1500 mg/L are potentially fatal due to neurological dysfunction [8, 9].

A distinctive characteristic of methanol poisoning is the delayed onset of symptoms. This latency period can be influenced by the concomitant consumption of ethanol, which modulates methanol metabolism [10]. Although the duration of this delay is variable, the progression of symptoms can be rapid. Initial symptoms, excluding visual disturbances, are non-specific, encompassing nausea, vomiting, and abdominal pain. Subsequent symptoms are attributed to acidosis resulting from the accumulation of formic and lactic acids.

The clinical course typically ranges from 6 to 36 h, frequently terminating in respiratory failure. Current therapeutic modalities include gastric lavage, ethanol therapy, inhibition of alcohol dehydrogenase via fomepizole, dialysis, alkalinization, and administration of cofactors, such as folate [10–12]. Despite advancements in treatment protocols, the mortality rate remains elevated, primarily due to challenges in achieving timely diagnosis.

Here, we report a patient with severe methanol intoxication presenting with metabolic acidosis, who developed diffuse cerebral edema manifested as pseudo-SAH on brain imaging and progressed to brain death within 3 days despite treatment.

## Case presentation

A 31-year-old Caucasian Iranian male was brought to the emergency department by emergency medical services (EMS) after being found unresponsive at home. According to family members, he had ingested approximately 1–2 L of homemade methanol 4–5 h prior to presentation. On EMS arrival, the patient was found sitting upright with vomitus on his face, a Glasgow Coma Scale (GCS) score of 3, gasping respirations, and an electrocardiographic heart rate of 32 beats per minute (bpm) with no palpable pulse, consistent with pulseless electrical activity. Oxygen saturation was 80% following endotracheal intubation.

Upon arrival at the emergency department (ED), the patient experienced cardiac arrest. Cardiopulmonary resuscitation (CPR) and epinephrine administration were initiated. After 10 min of CPR, a shockable rhythm (V-tach) was successfully converted to a stable sinus rhythm with a heart rate of 100 bpm. His Laboratory findings revealed severe metabolic acidosis (pH 7.15, HCO3 7.1 mmol/L, pCO2 20.3 mmHg), acute kidney injury (creatinine 3.4 mg/dL, urea 73 mg/dL, potassium 6.1 mmol/L, sodium 147 mmol/L), elevated liver function tests (Aspartate aminotransferase (AST) 366 U/L, Alanine aminotransferase (ALT) 146 U/L), leukocytosis (WBC 14.3 × 10^9/L), elevated lactate dehydrogenase (LDH 2195 U/L), elevated amylase (874 U/L), and markedly elevated creatine phosphokinase (CPK 16609 U/L). Urine analysis also confirmed methanol presence and his neurological examination revealed bilaterally mid-sized pupils that were reactive to light.

Following transfer to the intensive care unit (ICU), the patient underwent 5 h of hemodialysis. Due to persistent low oxygen saturation and coarse crackles on bilateral lung auscultation, a chest computed tomography (CT) scan was performed, confirming aspiration pneumonia and acute respiratory distress syndrome (ARDS), necessitating mechanical ventilation. The treatment regimen included dexamethasone (4 mg three times daily), meropenem (500 mg once daily), clindamycin (600 mg every 8 h), vitamin B12, folic acid, vitamin B6, and 20% serum mannitol (120 mL stat, followed by 60 mL four times daily). Post-dialysis, the metabolic acidosis was resolved (HCO3 12 mmol/L).

**Fig. 1:**
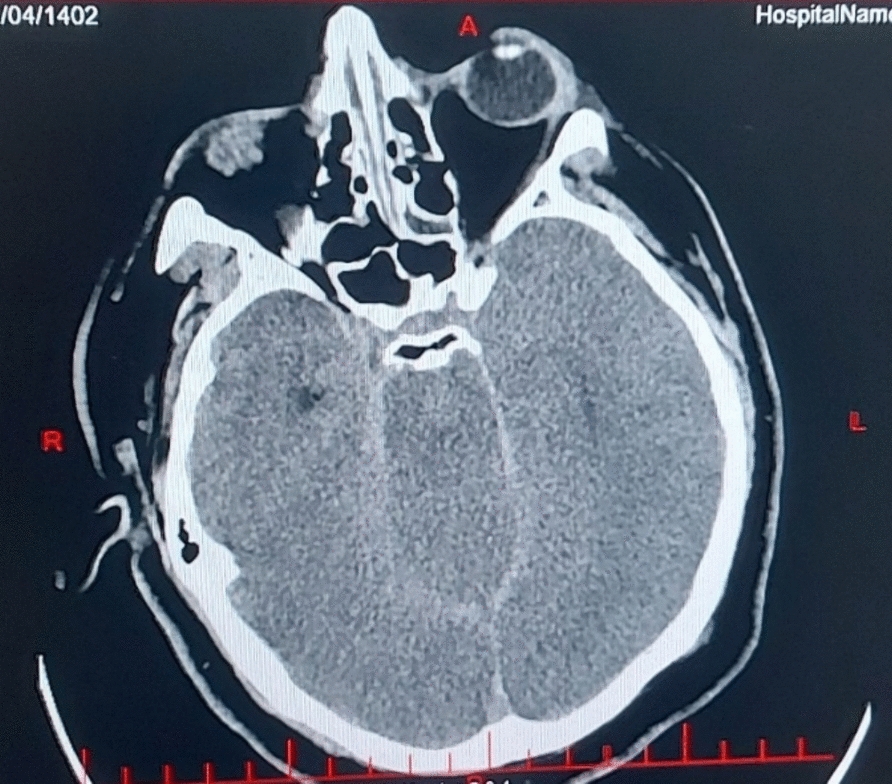
Brain CT demonstrating diffuse cerebral edema, loss of gray–white matter differentiation, and symmetric hyperdensity of the tentorium and falx consistent with pseudo-SAH

After 3 days of ICU admission with persistent coma, the patient developed fixed and dilated pupils, hyperemic optic discs, and complete absence of brainstem reflexes. Brain computed tomography revealed diffuse bilateral cerebral edema with marked loss of gray–white matter differentiation, more prominent in the cerebellum. Symmetric hyperdensities were noted along the tentorium cerebelli, falx cerebri, cerebral fissures, and basal cisterns, initially suggestive of subarachnoid hemorrhage. Following neurology consultation, these findings were reinterpreted as pseudo-SAH, reflecting severe cerebral edema. The CT scan also demonstrated transtentorial herniation with compression of the fourth ventricle and prepontine cisterns, without evidence of hydrocephalus (Fig. 1).

Neurological examination confirmed the absence of all brainstem reflexes, and the patient was declared brain dead after multidisciplinary consultation involving neurology, neurosurgery, and internal medicine. Brain death was attributed to diffuse cerebral edema and herniation secondary to hypoxic–ischemic brain injury caused by severe methanol intoxication, with pseudo-SAH representing a radiologic manifestation of this process.

## Discussion

Acute methanol poisoning, while rare, represents a serious type of alcohol-induced toxicity. The approximate lethal dose of methanol is given as 1 g/kg. Methanol itself is not considered to be directly toxic. However, its metabolism to formaldehyde by the liver and then to formic acid can be potentially lethal. There is a latent period of usually 6–24 h before the onset of symptoms. The clinical manifestations can vary from non-specific complaints of nausea, vomiting, and headache to potentially life-threatening conditions, such as high-anion-gap metabolic acidosis, visual disturbances, and CNS abnormalities. In the absence of appropriate treatment, mental status changes can progress to coma.

In the context of acute methanol poisoning, antidotal therapy aims at inhibiting the enzyme alcohol dehydrogenase (ADH) in order to prevent the formation of toxic metabolites, particularly formic acid, which has been implicated in causing severe metabolic acidosis and optic nerve damage. Fomepizole, an ADH inhibitor, has emerged as the antidote and should be initiated immediately in accordance with a strong suspicion of poisoning and the presence of high-anion-gap metabolic acidosis, without awaiting the confirmation of methanol levels in the blood. The antidotal regimen includes a loading dose of 15 mg/kg, followed by a dose of 10 mg/kg every 12 h, increasing to 15 mg/kg after 48 h, and continuing until the methanol levels are reduced below 30 mg/dL. In the context of hemodialysis, dose adjustment or continuous infusion at a rate of 1 mg/kg/h is indicated due to the extracorporeal clearance of the drug. Early antidotal therapy can prevent visual and neurological complications and, in some cases, may obviate the need for hemodialysis. Ethanol can also be used as an antidote in the absence of fomepizole, but this requires careful monitoring in order to maintain therapeutic levels of ethanol in the bloodstream (100–150 mg/dL) and is associated with a higher incidence of adverse effects [13, 14].

Management in this case was adapted according to the severity of the disease and the constraints of the institution. Quantitative serum methanol levels could not be performed; thus, the history of the case, the presence of significant high-anion-gap metabolic acidosis, and the progression of neurological symptoms were used for the diagnosis. Fomepizole was not included in the formulary of the hospital. Because of the severe condition of the patient (GCS of 3, renal failure, severe acidosis with a pH of 7.15), immediate dialysis was performed as the first line of treatment aimed at saving the life of the patient. Dialysis helps in the prompt removal of methanol and formate from the body while correcting life-threatening metabolic abnormalities.

There is also a link between methanol poisoning and ischemic or hemorrhagic cerebrovascular events, particularly involving the putamen and subcortical white matter. Under these conditions, pseudo-SAH is characterized as a radiological finding of severe cerebrovascular edema. Severe metabolic acidosis and hypoxia, as seen in methanol intoxication, can produce severe cerebrovascular edema, leading to imaging findings that mimic SAH. Pseudo-SAH can be differentiated from SAH by decreased attenuation values (30–42 HU vs. 60–70 HU), severe cerebrovascular edema (effacement of sulci and lack of gray–white matter differentiation), and significant contrast enhancement. Unlike SAH, pseudo-SAH does not involve intraventricular hemorrhage and is associated more often with severe brain injury or hypoxia–ischemia, as opposed to aneurysm rupture [15]. It is important to avoid such diagnostic errors, as they can lead to further, sometimes unnecessary, procedures, and, therefore, consideration of clinical data, biochemical abnormalities, and neurological signs is mandatory [12, 16].

The condition was first identified by Spiegel et al. (1986), who observed ten patients who died of diffuse cerebral edema without signs of SAH on autopsy [17]. Although the exact mechanism is unknown, severe cerebral edema can cause venous sinuses to compress, leading to poor venous drainage and hyperdense signs on CT scans, especially in low-density brain tissue [18]. Pseudo-SAH is most commonly observed in the basal cisterns and is associated with hypoxic–ischemic encephalopathy due to cardiac arrest or resuscitation. It can also be observed in meningitis, subdural hematomas, polycythemia, infarcts, and status epilepticus, as oxygen depletion is a key factor in pseudo-SAH [19, 20]. The key CT scan findings of pseudo-SAH include symmetric hyperdense tissue, soft tissue proliferation indicative of edema, and obliteration of basal cisterns [21].

During the COVID-19 pandemic, Pishbin et al. reported a 21-year-old male with seizures and severe metabolic acidosis (pH 6.7) following methanol ingestion. Brain CT showed diffuse cerebral edema, loss of gray–white differentiation, and basal cistern hyperattenuation mimicking SAH. Hounsfield unit measurements (~ 40 HU) confirmed pseudo-SAH. Despite fomepizole, sodium bicarbonate, and planned hemodialysis, the patient progressed to brain death within 24 h, highlighting the poor neurological prognosis associated with pseudo-SAH in methanol toxicity [16].

In a large retrospective study during the COVID-19-related methanol outbreak in Iran, Esmaeilian et al. (2023) analyzed 306 patients with confirmed methanol poisoning. Abnormal CT findings were observed in 15% of cases, most commonly putaminal hypodensity (11.1%), cerebellar nuclei hypodensity (8.2%), and diffuse cerebral edema (7.5%). Cerebellar nuclei involvement, diffuse cerebral edema, intracranial hemorrhage, and pseudo-SAH correlated strongly with poor outcomes, while nearly half of deceased patients initially had normal CT scans. Laboratory parameters including pH < 7.2, GCS < 8, renal failure, hyperglycemia, and hypoxemia correlated with abnormal imaging and mortality. The study reinforces that neuroimaging abnormalities in methanol toxicity primarily reflect advanced metabolic and hypoxic brain injury, emphasizing interpretation alongside clinical and laboratory severity markers [22].

## Conclusion

Pseudo-SAH represents a critical imaging pitfall in patients with severe methanol intoxication. It reflects advanced cerebral edema and hypoxic–ischemic injury rather than true hemorrhage. Awareness of this entity is essential to prevent misdiagnosis and unnecessary interventions. Early recognition on CT imaging, in conjunction with clinical and laboratory findings, may aid in appropriate prognostication and management.

## Data Availability

The data sets used during the current study are available from the corresponding author upon reasonable request.

## Abbreviations

CNS: Central nervous system
Pseudo-SAH: Pseudo-subarachnoid hemorrhage
EMS: Emergency medical services
GCS: Glasgow Coma Scale
bpm: Beats per minute
ED: Emergency department
CPR: Cardiopulmonary resuscitation
AST: Aspartate aminotransferase
ALT: Alanine aminotransferase
ICU: Intensive care unit
CT: Computed tomography
ARDS: Acute respiratory distress syndrome
SAH: Subarachnoid hemorrhage
ADH: Alcohol dehydrogenase
